# Enhancement of endothelial permeability by free fatty acid through lysosomal cathepsin B-mediated Nlrp3 inflammasome activation

**DOI:** 10.18632/oncotarget.12302

**Published:** 2016-09-28

**Authors:** Lei Wang, Yang Chen, Xiang Li, Youzhi Zhang, Erich Gulbins, Yang Zhang

**Affiliations:** ^1^ Institute of Hypertension, School of Medicine, Sun Yat-sen University, Guangzhou, China, 510080; ^2^ Department of Pharmacology and Toxicology, Virginia Commonwealth University, Richmond, VA 23298, USA; ^3^ Department of Pharmacological and Pharmaceutical Sciences, College of Pharmacy, University of Houston, Houston, TX 77204, USA; ^4^ Department of Molecular Biology, University of Duisburg-Essen, Essen, 45122, Germany

**Keywords:** free fatty acid, inflammasome, tight junction, cathepsin B, obesity

## Abstract

Obesity is an important risk factor for exacerbating chronic diseases such as cardiovascular disease and cancer. High serum level of saturated free fatty acids such as palmitate is an important contributor for obesity-induced diseases. Here, we examined the contribution of inflammasome activation in vascular cells to free fatty acid-induced endothelial dysfunction and vascular injury in obesity. Our findings demonstrated that high fat diet-induced impairment of vascular integrity and enhanced vascular permeability in the myocardium in mice were significantly attenuated by *Nlrp3* gene deletion. In microvascular endothelial cells (MVECs), palmitate markedly induces Nlrp3 inflammasome complex formation leading to caspase-1 activation and IL1β production. By fluorescence microscopy and flow cytometry, we observed that such palmitate-induced Nlrp3 inflammasome activated was accompanied by a reduction in inter-endothelial tight junction proteins ZO-1/ZO-2. Such palmitate-induced decrease of ZO-1/ZO-2 was also correlated with an increase in the permeability of endothelial monolayers treated with palmitates. Moreover, palmitate-induced alterations in ZO-1/ZO-2 or permeability were significantly reversed by an inflammasome activity inhibitor, YVAD, or a high mobility group box 1 (HMGB1) activity inhibitor glycyrrhizin. Lastly, blockade of cathepsin B with Ca-074Me significantly abolished palmitate-induced activation of Nlrp3 inflammasomes, down-regulation of ZO-1/ZO-2, and enhanced permeability in MVECs or their monolayers. Together, these data strongly suggest that activation of endothelial inflammasomes due to increased free fatty acids produces HMGB1, which disrupts inter-endothelial junctions and increases paracellular permeability of endothelium contributing to early onset of endothelial injury during obesity.

## INTRODUCTION

Obesity is an important risk factor for exacerbating chronic diseases, such as cardiovascular disease, type 2 diabetes, and neuronal damage [1]. Obesity has also been associated with certain types of cancers including endometrial, renal, colonic, and breast cancers [2]. Endothelial dysfunction is an early onset of vascular injury during metabolic disorders such as obesity [3–5]. It has been shown that loss of body weight can improve endothelial function and protect from coronary atherosclerotic injury. However, the precise mechanism initiating or triggering endothelial dysfunction and vascular injury associated with obesity remains elusive. It has been well accepted that the contribution of obesity to endothelial dysfunction is multifaceted. One of the important contributors is the high level of free fatty acids in the plasma. Intake of high fat diet containing mostly free fatty acids leads to the development of obesity. The saturated free fatty acid level in the plasma was elevated in humans with type 2 diabetes as well as in diabetic animals [6]. In this regard, recent studies have investigated whether saturated free fatty acids serve as important mediators to promote tissue inflammation and injury by activating toll-like receptors or inducing NF-κB-dependent inflammatory responses [7, 8]. However, most of the previous studies have focused on the effects of free fatty acids on their immune-modulatory roles in inflammatory cells such as macrophages [7, 8]. The direct actions of free fatty acids on the functions of vascular cells, particularly endothelial cells, have been relatively understudied.

Recent studies have highlighted a role of inflammasomes in sensing danger signaling and triggering inflammatory responses of tissues or organs [9–16]. The Nlrp3 inflammasome is one of the most important inflammasome isoforms involved in sterile inflammation induced by endogenous danger signals such as excessive extracellular ATP, crystals of monosodium urate or cholesterol crystals, and protein aggregates including β-amyloids [9, 10, 17, 18]. In immune cells, the Nlrp3 inflammasome monomer usually consists of three main subunits including the pattern recognition receptor Nlrp3, the adaptor protein ASC (apoptotic speck-containing protein with a CARD), and inactive executor pro-caspase-1 protein [19, 20]. Oligomerization of Nlrp3 monomers leads to the formation of a high-molecular-weight inflammasome complex, in which pro-caspase-1 is cleaved to its bioactive form and subsequently acts on over 120 protein substrates including precursors of pro-inflammatory cytokine interleukin-1β (IL-1β) and proteins in the glycosis pathways [21–23]. Recent studies have demonstrated that the Nlrp3 inflammasome is also present and functional in human and murine endothelial cells with similar subunits to the inflammasomes in immune cells [24–26]. Given the fact that vascular cells have much lower level of inflammatory responses upon stimulation compared to that in immune cells, it is plausible that the inflammasomes in vascular cells may have functions other than stimulating classical cytokine-mediated inflammatory responses found typically in immune cells. It is well documented that endothelial barrier dysfunction and hyperpermeability are the early events that ultimately lead to vascular inflammation and injury. In this study, we tested the hypothesis that Nlrp3 inflammasome activation in endothelial cells could be a triggering mechanism elicited by free fatty acids that contributes to the development of vasculopathy in obesity.

## RESULTS

### Nlrp3 gene deletion prevents high fat diet-induced vascular hyperpermeability in the myocardium in mice

**Figure 1:**
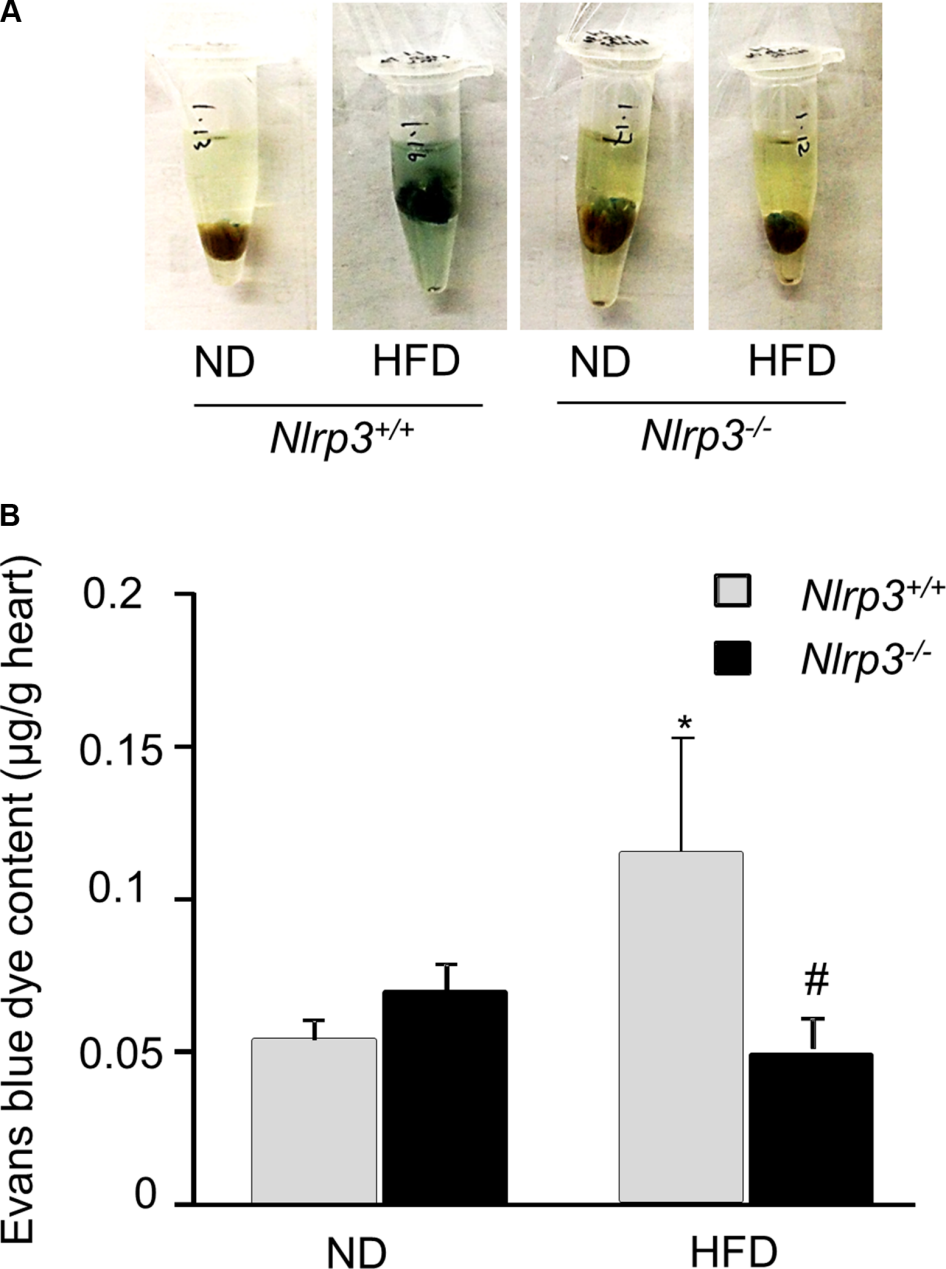
Nlrp3 gene deletion prevents high fat diet-induced vascular hyperpermeability in the myocardium of mice Mice (*Nlrp3*^+/+^ or *Nlrp3*^−/−^) were fed with normal diet (ND) or high fat diet (HFD) and then the vascular permeability of mouse hearts to Evans blue dyes was assessed as described in Methods. (**A**) Representative images of hearts before extraction and (**B**) summarized data show the effects of HFD on the concentration of Evans blue dyes in mouse hearts isolated from Nlrp3^+/+^ or Nlrp3^−/−^ mice (*n* = 5–8). *P < 0.05 vs. *Nlrp3*^+/+^ ND; ^#^*P* < 0.05 vs. *Nlrp3*^+/+^ with HFD.

To investigate the protective effect of *Nlrp3* gene deletion on high fat diet-induced vascular leakage in mouse heart *in vivo*, the mice were intravenously injected with Evans blue dye and leakage of Evans blue dye from plasma into the interstitial space was quantified. As shown in Figure 1, *Nlrp3*^+/+^ mice fed with high fat diet had significantly higher vascular permeability to intravenously injected Evans blue dye in the heart compared to that of *Nlrp3*^+/+^ mice fed with normal diet. *Nlrp3*^−/−^ mice had similar vascular permeability to Evans blue dye compared to *Nlrp3*^+/+^ mice when both mice were fed normal diet. However, *Nlrp3*^−/−^ mice fed with high fat diet had significantly reduced vascular permeability compared to that of *Nlrp3*^+/+^ mice fed with high fat diet. Thus, our findings demonstrated that high fat diet-induced impairment of vascular integrity was significantly attenuated when *Nlrp3* gene is deleted.

### Formation and activation of Nlrp3 inflammasomes in MVECs upon palmitate stimulation

**Figure 2:**
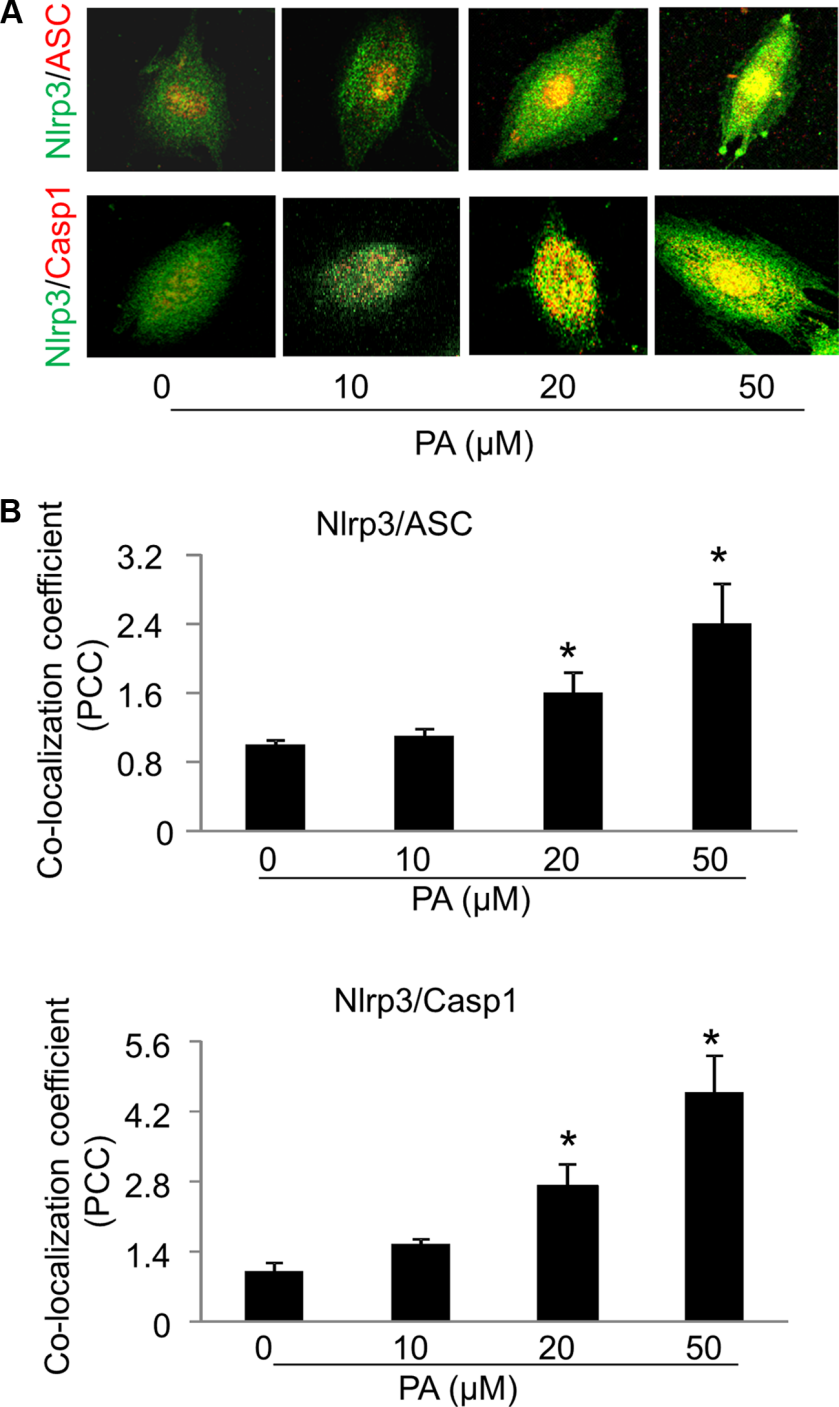
Palmitate increases the formation of Nlrp3 inflammasome complexes in MVECs (**A**) Representative confocal fluorescence images depict the effect of palmitate (PA: 0–50 μM; 24 hours) on the co-localization of Nlrp3 with ASC or caspase-1 (casp1). (**B**) Summarized data show the colocalization efficiency of Nlrp3 with ASC (*n* = 6–8) or Nlrp3 with caspase-1 (*n* = 3–6). *P < 0.05 versus control group.

**Figure 3:**
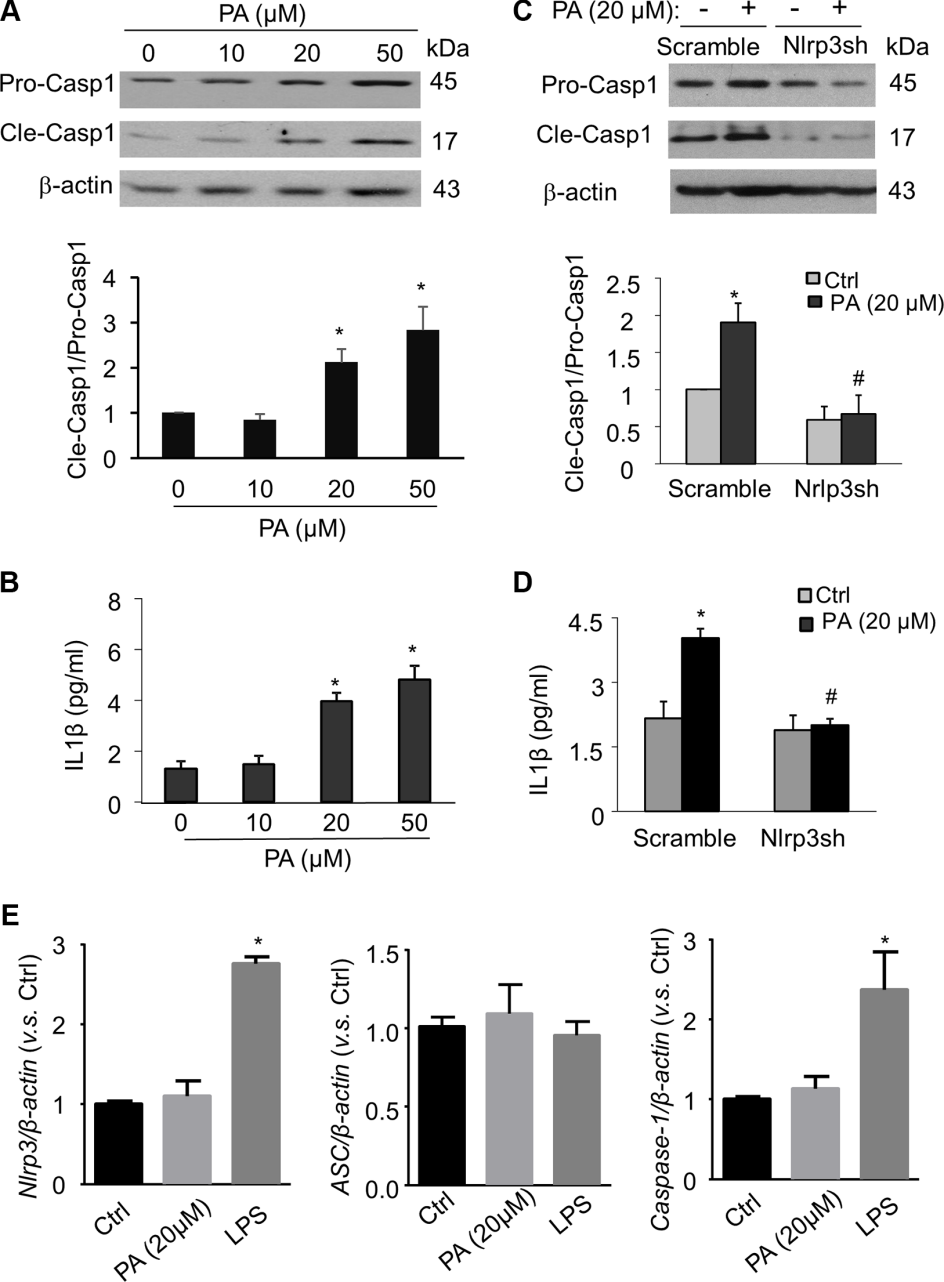
Palmitate activates Nlrp3 inflammasomes in MVECs (**A**) Representative Western blot documents and summarized data show the effects of palmitate (PA: 0–50 μM; 24 hours) on pro-caspase-1 (Pro-casp1) and cleaved caspase-1 (Cle-casp1) expression in MVECs (*n* = 4). (**B**) Summary of data for IL-1β production compared with untreated control (*n* = 4–6). *P < 0.05 versus control group. (**C** and **D**) Representative Western blot documents and summarized data show the effects of scramble or Nlrp3 gene silencing on caspase-1 processing (*n* = 3) and IL-1β production (*n* = 3–6) in MVECs stimulated with palmitate (20 μM; 24 hours). *P < 0.05 versus control group. *P < 0.05 vs. Scramble Ctrl; ^#^*P* < 0.05 vs. Scramble with PA. (**E**) Summarized data the effects of palmitate (20 μM; 24 hours) or lipopolysaccharides (LPS, 1 μg/ml) on the relative mRNA levels of Nlrp3, ASC, and caspase-1 genes in MVECs. *P < 0.05 vs. Ctrl.

We next investigated whether increased level of free fatty acid, a typical danger signal associated with obesity and type 2 diabetes, can elicit Nlrp3 inflammasomes in endothelial cells and subsequently contribute to the development of endothelial dysfunction and vascular injury. Palmitate has been shown as one of the most abundant saturated fatty acid in the plasma and its plasma level is substantially elevated following high fat diet [7, 27]. In cultured MVECs, we examined whether palmitate could trigger the formation and activation of Nlrp3 inflammasome complexes by analyzing the co-localization of Nlrp3 inflammasome components, the cleavage of pro-caspase-1 to activate caspase-1, and the production of IL-1β. As shown in Figure 2, palmitate increased the co-localization between Nlrp3 (green) and ASC (red) or Nlrp3 (green) and caspase-1 (red) in MVECs as shown by increased yellow staining and co-localization coefficient. The maximal co-localization for both Nlrp3/ASC and Nlrp3/caspase-1 was observed with palmitate concentration at 50 μM. Formation of Nlrp3 inflammasome complexes results in the cleavage of pro-caspase-1 protein to its bioactive form, which in turn binds to and cleaves its substrates such as pro-interleukin 1 β (IL-1β). Consistent with the confocal findings for the formation of Nlrp3 inflammasome complex, we also demonstrated that palmitate increased expression of cleaved caspase-1 and IL-1β production (Figure 3A–3B). Such palmitate-induced caspase-1 activation and IL-1β production were abolished in MVECs with Nlrp3 gene silencing (Figure 3C–3D). This result confirms that Nlrp3 is the primary inflammasome isoform to be activated by palmitate stimulation. 20 μM palmitate was used as an optimal dose to activate inflammasome as at 50 μM palmitate starts to induce cell death in MVECs. Lipopolysaccharide (LPS) was reported to activate inflammasome gene expression in mammalian cells [28]. Here, we demonstrate that LPS from *E.coli*, but not palmitate, stimulates Nlrp3 and caspase-1 gene expression in MVECs suggesting that the activation of Nlrp3 inflammasomes by palmitate was not associated with up-regulation of the mRNA levels of inflammasome components (Figure 3E).

### Effects of caspase-1 inhibition on palmitate-induced changes in tight junction proteins and endothelial permeability in MVECs

**Figure 4:**
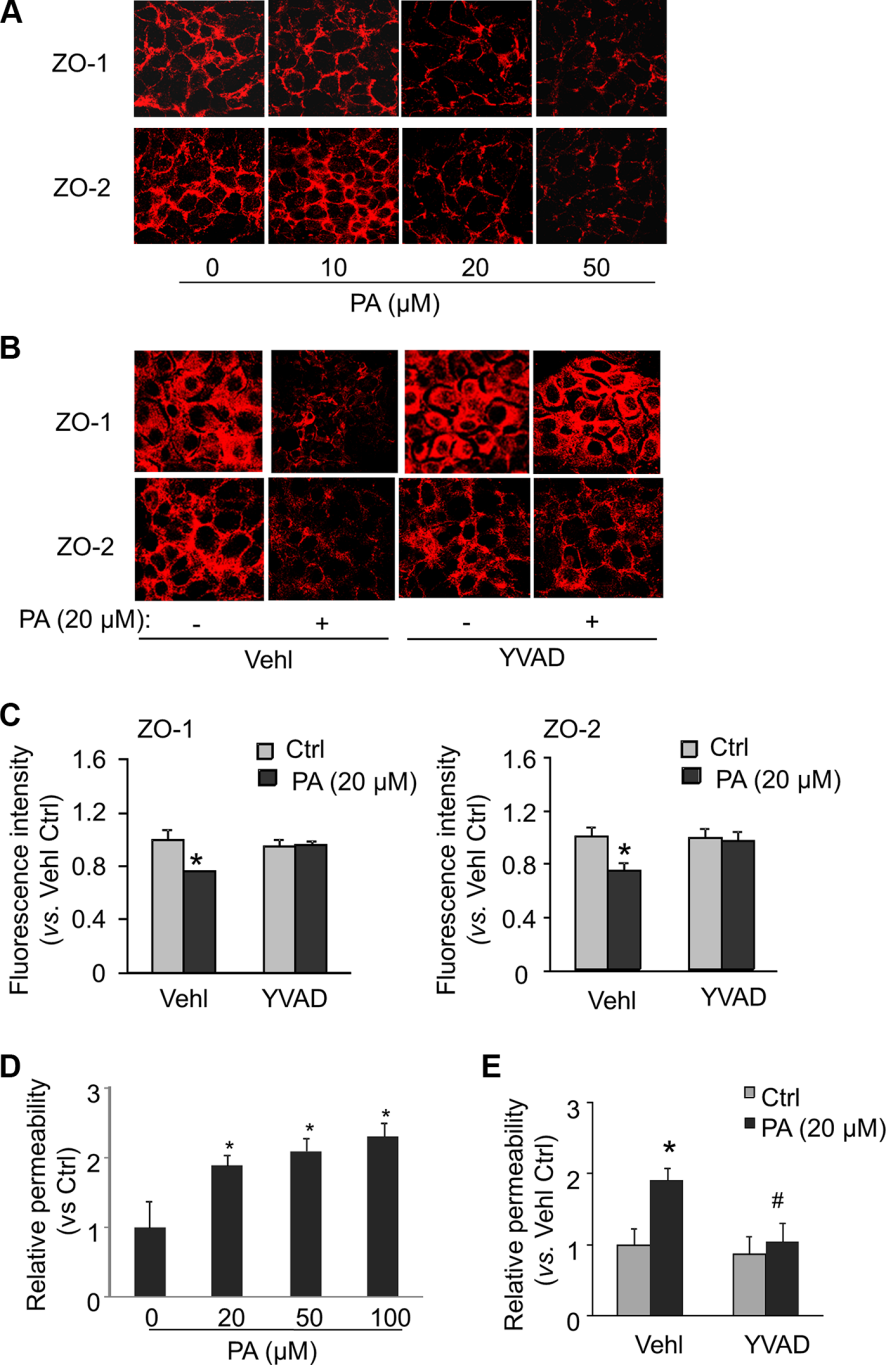
Dependence of palmitate-induced tight junction disruption and enhanced permeability on caspase-1 MVECs were stimulated with different concentrations of palmitate (PA: 0–50 μM) or with 20 μM palmitate in the presence of PBS (Vehl for vehicle) or caspase-1 inhibitor Ac-YVAD-CMK (YVAD: 10 g/ml) for 24 hours. (**A** and **B**) Representative fluorescence images show the cell membrane fluorescence of ZO-1 and ZO-2 from at least three independent experiments. (**C**) The protein expression of ZO-1 and ZO-2 were quantified by flow cytometry. Summarized data show the mean fluorescence intensity of ZO-1 or ZO-2 (*n* = 5–8). (**D** and **E**) MVECs on inserts of transwells were treated as above. Summarized data show the relative permeability of endothelial monolayers in the inserts for FITC-dextran (*n* = 4–5). *P < 0.05 versus Vehl Ctrl; ^#^*P* < 0.05 vs. Vehl with PA.

The paracellular permeability of endothelium depends on the integrity of protein complexes called inter-endothelial junctions. We examined whether palmitate-induced Nlrp3 inflammasome activation could cause disassembly of junction proteins in cultured endothelial cells *in vitro*. Tight junction proteins ZO-1/ZO-2 are essential endothelial cell membrane-associated proteins in establishing and organizing the paracellular barrier and barrier-based selectivity of membrane. Down-regulation of tight junction proteins ZO-1/ZO-2 is well known as a marker event of tight junction disassembly leading to tight junction disruption and enhanced paracellular permeability. By confocal microscopy, we found that palmitate significantly decreased the expression of ZO-1/ZO-2 on the membrane, particularly in the cell-cell contact regions in the monolayers of MVECs (Figure 4A). Down-regulation of ZO-1/ZO-2 induced by palmitate was prevented bin cells treated with caspase-1 inhibitor Ac-YVAD-CMK (YVAD, Cayman Chemical). The surface expression of ZO-1/ZO-2 in MVECs was examined by flow cytometry analysis. It was found that palmitate decreased the surface expression of ZO-1/ZO-2 and such effect of palmitate on surface expression of ZO-1/ZO-2 was also blocked by caspase-1 inhibition using Ac-YVAD-CMK. Therefore, these results indicate that palmitate-induced decreases in ZO-1/ZO-2 protein expressions are attributed to the decreases in their surface expression, which is dependent on caspase-1 activation. Consistent with the findings in ZO-1/2 expression, we also observed a dose-dependent increase in the relative permeability of endothelial cell monolayers by palmitate. Such palmitate-induced increase in permeability was prevented in cells treated with caspase-1 inhibitor Ac-YVAD-CMK.

### HMGB1 inhibition prevents palmitate-induced changes in tight junction proteins and endothelial permeability in MVECs

**Figure 5:**
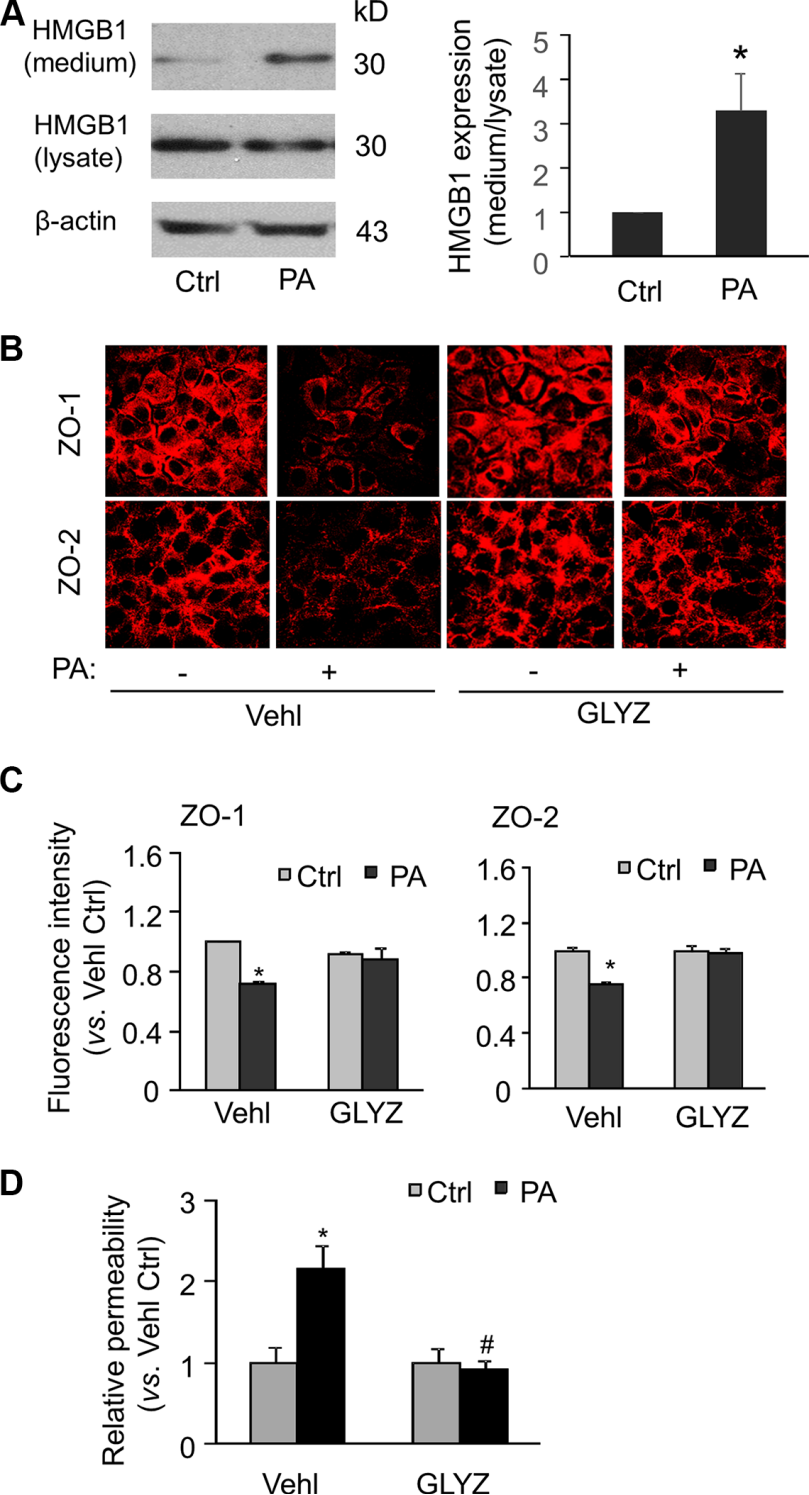
HMGB1 inhibitor glycyrrhizin abolishes palmitate-induced tight junction disruption and enhanced permeability in MVECs MVECs were stimulated with 20 μM palmitate in the presence of PBS (Vehl for vehicle) or HMGB1 inhibitor glycyrrhizin (GLYZ, 130 μM). (**A**) Western blot documents and summarized data showing the expression of HMGB1 in either cell culture medium (Medium) or cell homogenates (lysate) (*n* = 4). (**B**) Representative fluorescence images show the cell membrane fluorescence of ZO-1 and ZO-2 from at least three independent experiments. (**C**) The protein expression of ZO-1 and ZO-2 were quantified by flow cytometry. Summarized data show the mean fluorescence intensity of ZO-1 or ZO-2 (*n* = 4). (**D**) Summarized data show the relative permeability of endothelial monolayers in the inserts for FITC-dextran (*n* = 4–8). *P < 0.05 versus Vehl Ctrl; ^#^*P* < 0.05 vs. Vehl with PA.

HMGB1 is a conserved nuclear protein involved in the maintaining of DNA structure in the nucleus. In another aspect, HMGB1 has also been shown to be released into extracellular space upon Nlrp3 inflammasome activation and can serve as a novel permeability factor on vascular endothelium *in vitro* and *in vivo* [29, 30]. Here, we explored whether the HMGB1 is involved in palmitate-induced changes in tight junction proteins and endothelial permeability. As shown in Figure 5A, Western blot analysis demonstrated that palmitate significantly increased the release of HMGB1 protein from MVECs to the culture media. Confocal microscopy and flow cytometry analyses demonstrated that the decreases in the surface expression of ZO-1/ZO-2 by palmitate stimulation were blocked by glycyrrhizin, a functional inhibitor of HMGB1 activity (Figure 5B and 5C). Consistently, the increase in the endothelial permeability induced by palmitate was also inhibited by glycyrrhizin (Figure 5D). Together, these results suggest that palmitate-induced disruption of tight junction protein and endothelial permeability is dependent on HMGB1 release by endothelial cells.

### Effects of cathepsin B inhibition on palmitate-induced inflammasome activation, tight junction disruption, and permeability in MVECs

**Figure 6:**
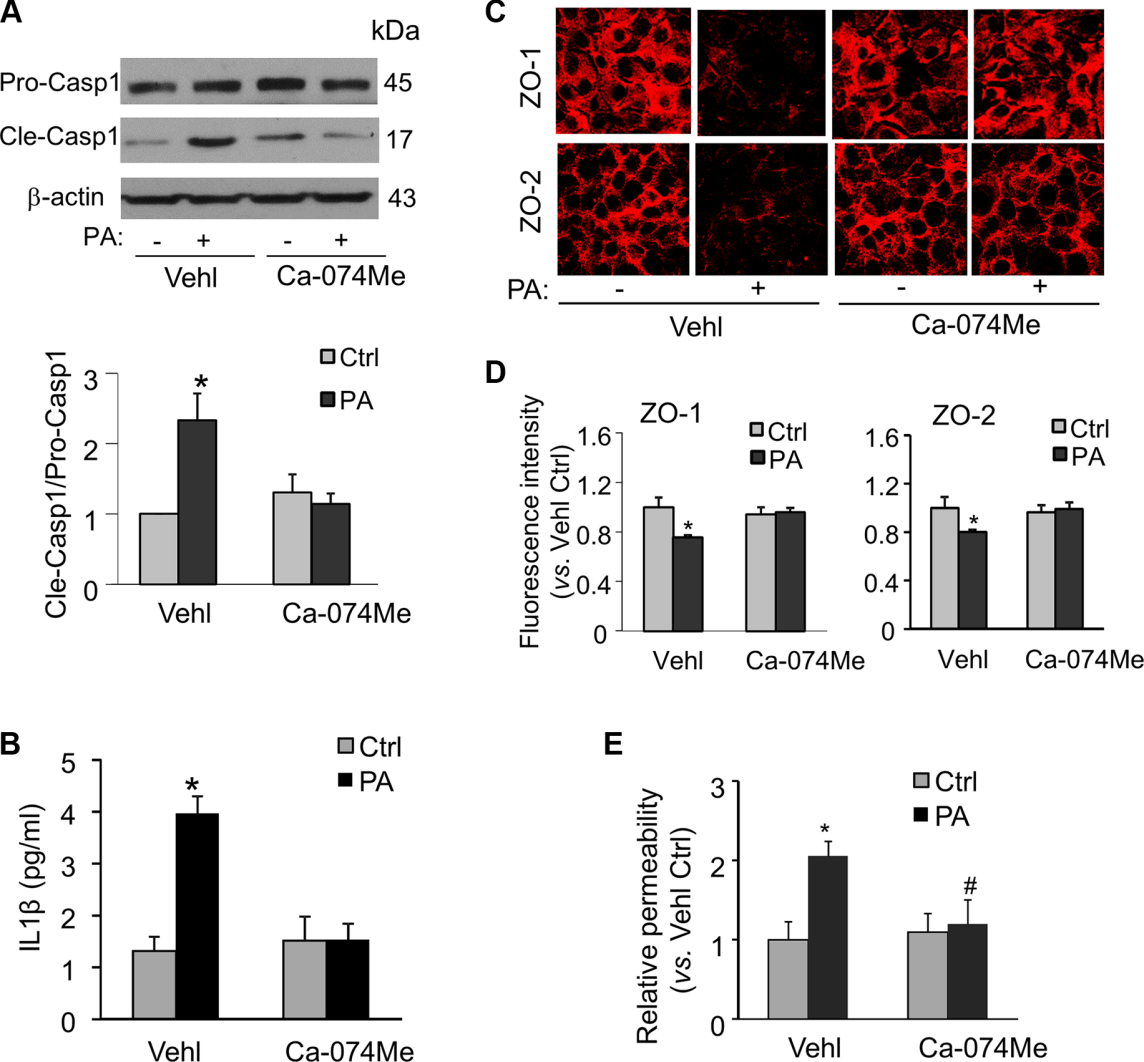
Inhibition of cathepsin B activity abolishes palmitate-induced Nlrp3 inflammasome activation, tight junction disruption, and enhanced permeability in MVECs MVECs were stimulated with 20 μM palmitate in the presence of PBS (Vehl for vehicle) or cathepsin B inhibitor Ca-074Me (5 μM). (**A**) Western blot documents and summarized data showing the pro-caspase-1 (Pro-casp1) and cleaved caspase-1 (Cle-casp1) expression. (*n* = 4). (**B**) Summary of data for IL-1β production compared with untreated control (*n* = 5–8). (**C**) Representative fluorescence images show the cell membrane fluorescence of ZO-1 and ZO-2 from at least three independent experiments. (**D**) The protein expression of ZO-1 and ZO-2 were quantified by flow cytometry. Summarized data show the mean fluorescence intensity of ZO-1 or ZO-2 (*n* = 8). (**E**) Summarized data show the relative permeability of endothelial monolayers in the inserts for FITC-dextran (*n* = 3–4). *P < 0.05 versus Vehl Ctrl; ^#^*P* < 0.05 vs. Vehl with PA.

Recent studies have demonstrated that palmitate activates the Nlrp3 inflammasome in bone marrow macrophages through a mechanism that involves the lysosome and its protease cathepsin B [31]. Here, we tested whether palmitate could also stimulate Nlrp3 inflammasomes in a cathepsin B-dependent manner in endothelial cells. As shown in Figure 6A and 6B, palmitate-induced caspase-1 activation and IL-1β production were both prevented in MVECs treated with cathepsin B inhibitor Ca-074Me (Sigma). Furthermore, inhibition of cathepsin B using Ca-074Me also abolished the palmitate-induced disruption of tight junction proteins ZO-1/ZO-2 (Figure 6C and 6D) and enhancement of endothelial permeability in MVECs (Figure 6E). These results suggest that cathepsin B-dependent Nlrp3 inflammasome activation contributes to palmitate-induced tight junction disruption and hyperpermeability in MVECs.

## DISCUSSION

The major goal of this study was to determine whether endothelial hyperpermeability is associated with endothelial Nlrp3 inflammasome activation induced by increased level of saturated free fatty acid, an important danger signal associated with obesity. Our findings provide mechanistic insights regarding the activation of the inflammasome by saturated free fatty acids through a lysosomal cathepsin B signaling. The inflammasome-dependent factors such as HMGB1 target endothelial cells via autocrine or paracrine actions to reduce the expression of inter-endothelial tight junction proteins that are essential for maintaining the endothelial barrier function and vascular integrity.

Recent studies have demonstrated a role of Nlrp3 inflammasome in sensing obesity-associated danger signals such as saturated free fatty acids and lipotoxicity-associated increases in intracellular ceramide to induce caspase-1 cleavage in macrophages and adipose tissue that contributes to obesity-induced inflammation and insulin resistance [7, 32]. It should be noted that in these studies, the Nlrp3 inflammasome activity was observed at a relatively late stage of obesity or metabolic stress as the mice were treated with high fat diet for more than twelve weeks. In another aspect, six-week high fat diet treatment activated endothelial Nlrp3 inflammasomes and increased T cell transmigration in coronary arteries in mice [33]. The present study, for the first time, provided direct evidence showing that six-week high fat diet treatment enhanced the vascular permeability in the myocardium *in vivo*, which was ameliorated by *Nlrp3* gene deletion. Together, these data imply that during the early stage of obesity endothelial inflammasome activation could initiate endothelial barrier dysfunction leading to vascular hyperpermeability, which facilitates inflammatory cells infiltration through endothelium contributing to vascular inflammation and injury in obesity.

Accumulating evidence indicate that free fatty acid-induced endothelial inflammation and dysfunction are importantly involved in the pathogenesis of vascular diseases associated with obesity and related metabolic disorders [1, 27]. The sixteen-carbon palmitate is one of the three major free fatty acids that were found in human plasma and has been shown to activate Nlrp3 inflammasomes in macrophages and human endothelial cells [7, 26]. Consistently, our data demonstrated that palmitate as low as 20 μM can stimulate Nlrp3 inflammasomes as characterized by increased formation of Nlrp3 inflammasome complexes, caspase-1 activation, and IL-1β production. The concentrations of palmitate in humans range from 10 to 50 μM [34]. Thus, our observation suggests that mouse endothelial cells are more sensitive to palmitate-induced endothelial cell activation. Further, our data (Figure 3E) show that palmitate has no effects on the transcription level of inflammasome genes including Nlrp3, ASC, and caspase-1 in MVECs implicating that palmitate-induced Nlrp3 inflammasome activation in murine endothelial cells is not associated with transcriptional upregulation of inflammasome components. In contrast, LPS, a toll-like receptor 4 (TLR4) agonist, increase Nlrp3 and caspase-1 mRNA levels suggesting that palmitate does not activate TLR4 in MVECs. Further, our finding seems to be inconsistent with a recent study demonstrating that palmitate at 100 μM increases the protein expression of Nlrp3 and caspase-1 in human umbilical vein endothelial cells [26]. The discrepancy between these studies is unknown and may be due to the sources or species of endothelial cells (umbilical vein vs. microvascular; human vs. murine). Nonetheless, these studies support the notion that free fatty acid could instigate inflammasomes in vascular endothelial cells causing endothelial cell dysfunction or injury independent of immune cells.

Our findings reveal that free fatty acid-induced endothelial hyperpermeability is associated with inflammasome-dependent tight junction disruption. Vascular endothelium is a monolayer of endothelial cells which forms interface and serves as a barrier between the lumen and the vessel wall. In physiological conditions, the endothelium adaptively alters its functional state in response to various blood-borne or local stimuli that contributes to vascular homeostasis. Conversely, in conditions such as obesity and dyslipidemia, endothelium can be excessively stimulated and maladaptively modulate its functions resulting in localized damages in the vascular wall [35]. Endothelial barrier dysfunction is characterized by enhanced endothelial permeability to macromolecules or cells in plasma such as lipoproteins and leukocytes. It has been well accepted that loss of the integrity of inter-endothelial tight junctions contributes to enhanced paracellular endothelial permeability. Plasma proteins including albumin and visfatin can impair renal tubular or endothelial tight junctions via activation of Nlrp3 inflammasomes [33, 36]. Consistent with these previous studies, the present study finds that palmitate treatment induces decreases in typical tight junction proteins ZO-1/ZO-2 and increases in permeability to dextrans in MVECs, which are prevented by inhibiting caspase-1, a key and executive component of Nlrp3 inflammasome (Figure 4). Similar inhibitory effects were found by the presence of HMGB1 inhibitor (Figure 5), suggesting that HMGB1 is downstream of Nlrp3 inflammasome signaling. Recent studies by ours and others have suggested that HMGB1 can serve as a novel permeability factor for epithelial or endothelial barrier in intestine, lung or heart possibly via HMGB1-RAGE (receptor for advanced glycation endproducts) signaling axis [29, 30, 33]. Thus, palmitate-enhanced Nlrp3 inflammasome activity leads to impairment of endothelial barrier, presumably by a release of HMGB1 that promotes tight junction disruption.

The present study further identifies the mechanism by which free fatty acid activates inflammasome leading to endothelial hyperpermeability. Cathepsin B is a lysosomal protease primarily involved in the degradation of lysosomal proteins to maintain cellular metabolism. Cathepsin B activation is implicated in a variety of pathological processes, including lipotoxicity, apoptosis, and inflammation [37]. Lysosomal cathepsin B is activated upstream of Nlrp3 inflammasome activation and IL-1β secretion in response to a variety of stimuli including cholesterol crystals, *Trypanosoma cruzi*, and *lactobacillus casei* cell wall fragments [18, 25, 38]. Previous studies demonstrated that free fatty acid treatment of hepatocytes resulted in lysosomal destabilization with release of cathepsin B into the cytosol, which could lead to mitochondrial dysfunction and reactive oxygen species (ROS) production [39, 40]. Mitochondrial-derived ROS can serve as a potent activator for Nlrp3 inflammasome via thioredoxin-interacting protein [19]. Consistent with these previous studies, our data demonstrate that palmitate-induced caspase-1 activity and IL-1β production were blocked by cathepsin B inhibitor Ca-074Me (Figure 6A–6B). These findings, for the first time, demonstrate that cathepsin B activation by free fatty acid leads to Nlrp3 inflammasome activation in endothelial cells. The present study did not attempt to further dissect the interplay between cathepsin B and ROS in the context of inflammasome activation. More importantly, we explored that the decreased tight junction expression and enhanced endothelial permeability by palmitate were attenuated by the presence of cathepsin B inhibitor, suggesting that endothelial hyperpermeability by free fatty acid could be ameliorated through inhibiting cathepsin B-Nlrp3 inflammasome pathway. Thus, targeting cathepsin B/Nlrp3 inflammasome signaling axis could be a potential therapeutical strategy for treating cardiovascular diseases in obesity.

In summary, this work has studied the molecular mechanisms how high fat diet induces inflammasome-dependent endothelial hyperpermeability. Our data suggest that elevated free fatty acids caused by a high fat diet can activate the Nlrp3 inflammasome in endothelial cells via cathepsin B signaling pathway that has not been appreciated previously. HMGB1 induced by free fatty acids promotes the tight junction disruption and endothelial hyperpermeability, ultimately contributing to the endothelial dysfunction and injury during obesity. Obesity has been shown to increase progression and metastasis of breast cancer [41]. In this respect, high-fat diets promote metastasis of ID8 ovarian cancer cell and 4T1 murine mammary carcinoma cells in murine diet-induced obesity models [42, 43]. Thus, our findings provide novel insights that free fatty acid-induced endothelial hyperpermeability via inflammasome activation may facilitate cancer cells to extravasate endothelial barrier thereby contributing to metastasis in obesity.

## MATERIALS AND METHODS

### Animal procedures

Eight-week-old male C57BL/6J wild-type (Nlrp3^+/+^) and Nlrp3^−/−^ mice were used in the present study (The Jackson Laboratory). Nlrp3 knockout (*Nlrp3*^−/−^) and wild type (*Nlrp3*^+/+^) mice were genotyped following the protocol by the vendor. For 6 weeks, mice were fed either a normal diet or a high fat diet (60 kcal % fat; Research Diets, USA) as reported previously [33, 44]. All protocols were approved by the Institutional Animal Care and Use Committee of Virginia Commonwealth University. After 6 weeks, mice were sacrificed and heart tissues were harvested for immunofluorescence or biochemical examinations.

### Vascular permeability assay

Assessment of vascular permeability in mouse hearts was performed as described previously [45]. Mice were anesthetized using isoflurane and intravenously injected with 30 mg/kg of Evans blue solution. After 2 h, the mice were sacrificed by cervical dislocation and injected with PBS through ventricle to remove intravascular Evans blue solution. The Evans blue dye was eluted from the dissected heart by incubation with formamide at 60°C for 2 days. The amount of dye was quantitated by spectrophotometry at 620 nm.

### Cell culture

The mouse microvascular endothelial cell (MVEC) line EOMA was purchased from ATCC and cultured as we recently described [25]. MVECs were cultured in Dulbecco's modified Eagle's medium (DMEM) (Gibco, USA), containing 10% of fetal bovine serum (Gibco, USA) and 1% penicillin–streptomycin (Gibco, USA). The cells were ured in a humidified incubator at mixture at 37°C with 5% CO_2_ and 95% air. Cells were passaged by trypsinization (Trypsin/EDTA; Sigma, USA), followed by dilution in DMEM medium containing 10% fetal bovine serum.

### Sodium palmitate solution

Sodium palmitate was purchased from Sigma. Sodium palmitate solution was prepared as previously described [7]. Briefly, palmitate was dissolved in warm 50% ethanol at 55°C to make a 200 mmol/L palmitate stock solution. To make 5 mmol/L palmitate/10% fatty acid-free bovine serum albumin (BSA) solution, 25 μl palmitate stock solution was added into 10% fatty acid-free BSA solution. The palmitate and BSA mixture was incubated for 5 min at 55°C to obtain a homogeneous mixture.

### Immunofluorescence microscopic analysis

Cells were grown on eight-well chamber slides and then treated as indicated and then fixed in 4% paraformaldehyde for 15 minutes. Cells were washed in phosphate-buffer saline (PBS) and cells were incubated for 2 hours at 4°C with incubated with rabbit and/or mouse anti-Nlrp3 (1:500, Abcam), anti-ASC (1:500, Invitrogen, Abcam), anti-caspase 1 (1:1000; Abcam), anti-ZO-1 (1:1000; Invitrogen), and anti-ZO-2 (1:1000; Invitrogen). Double immunofluorescent staining was performed by incubating slides with Alexa Fluor 488 or Alexa Fluor 555-labeled secondary antibody (1:100, Invitrogen) for 1 hour at room temperature. The slices were visualized through sequentially scanning on an Olympus laser scanning confocal microscope (Fluoview FV1000, Olympus, Japan). Colocalization was analyzed by Image Pro Plus software, and the co-localization coefficient was represented by Pearson's correlation coefficient.

### Immunoblotting

Cells were washed twice with ice-cold PBS and homogenized in ice-cold HEPES buffer containing 25 mM Na-HEPES, 255 mM sucrose, 1 mM EDTA, and 0.1 mM phenylmethylsulfony1 fluoride (pH 7.4). After centrifugation at 1000 × g for 10 min at 4°C, the supernatants containing the membrane protein and cytosolic components, termed homogenates, were frozen in liquid N_2_, and stored at −80°C until use. Cell homogenates were denatured with reducing Laemmli SDS-sample buffer and boiled for 5 min. Samples were run on SDS-PAGE gel, transferred into PVDF membrane and blocked. The membranes were probed with primary antibodies (Abcam, 1:1000) against caspase-1, HMGB1, or β-actin overnight at 4°C followed by incubation with secondary antibody, and then conjugated to horseradish peroxidase-labeled immunoglobulin G. The immunoreactive bands were enhanced by chemiluminescence methods and imaged on Kodak Omat film. β-actin served as a loading control. The intensity of the bands was quantified by densitometry.

### Nucleofection

Transfection of shRNA plasmids was performed using a 4D Nucleofector X-Unit (Lonza) according to the manufacturer's instructions as previously described [46]. The plasmid encoding shRNA for mouse Nlrp3 gene was obtained from Origene (#TG510752, Rockville, MD). Briefly, MVECs were tyrpsinized and centrifuged at 80′ g for 10 min. The cell pelletet was resuspended in 100 mL SF Nucleofection solution (Lonza) for Nucleofection (with the program code DS198). The program was chosen based on the fact that over 80% of Nucleofected cells were positive for GFP control plasmid as analyzed by flow cytometry (GUAVA, Hayward, CA). For each Nucleofection sample (2 × 10^6^ cells/sample), 2 μg plasmid DNA was added in 100 μL SF Nucleofection solution. After Nucleofection, cells were cultured in DMEM medium for 24 hours.

### Real-time reverse transcription polymerase chain reaction (RT-PCR)

Total RNA from cells was extracted with Aurum Total RNA isolation kit (Bio-Rad) according to the manufacturer's protocol. One-microgram aliquots of total RNA from each sample were reverse-transcribed into cDNA by using a first-strand cDNA synthesis kit (Bio-Rad). Equal amounts of the reverse transcriptional products were subjected to PCR amplification PrimePCR™ SYBR^®^ Green Assay on a CFX Connect Real-Time PCR Detection System (Bio-Rad). The primers for detecting Nlrp3 (Bio-Rad#qMmuCID0010647), ASC (Bio-Rad#qMmuCED0047869), and caspase-1 (qMmuCID0026983) genes were purchased from Bio-Rad (primers were certified by Bio-Rad); the primers for β-actin: sense TCGCTGCGCTGGTCGTC, antisense GGCCTCGTCACCCACATAGGA.

### Flow cytometry analysis

Cells were cultured in 24-well culture plates and treated as indicated. Then cells were washed in PBS containing 1% Tween 20 (PBST) in 5 min for three times at 4°C. Cells were incubated with 5% Donkey serum in PBST for 30 min and then incubated with rabbit anti-ZO-1 (1:1000, Invitrogen) or anti-ZO-2 (1:1000, Invitrogen) for 1 h at 4°C. After incubation with antibodies, the cells were washed in PBST in 5 min for three times and then stained for another 30 min with Alexa Fluor 555-labeled secondary antibody (1:1000, Invitrogen). After another three times 5-min washes in PBST, the cells were trypsinized by 100 μl 2× trypsine for 1 min and terminated by 500 μl DMEM media. Then the cells in each well were suspended and analyzed for red fluorescent signal by flow cytometry analysis using a flow cytometer (GUAVA, Hayward, CA, USA).

### Endothelial permeability

MVECs were cultured in 24-well transwell plates and treated as indicated for 24 hr. The transwell inserts were moved into non-used wells with 200 μl fresh media. 100 μl Fluorescein isothiocyanate (FITC)–dextran (10 KDa, Invitrogen) solution was added into each insert and the plate was incubated at 37°C for 2 hours to allow fluorescein molecules flow through the endothelial cell monolayer. The inserts were then removed and fluorescent intensity in each well was determined at excitation/emission of 485/530 nm using a fluorescent microplate reader (FL × 800, BIO-TEK Instruments). The arbitrary fluorescence intensity was used to calculate the relative permeability.

### IL-1β production

After treatment, the cell supernatant was collected and IL-1β production was measured by a commercially available ELISA Kit (R&D System, Minneapolis, MN) according to the protocol described by the manufacturer.

### Statistics

Data are presented as means ± SE. Significant differences between and within multiple groups were examined using ANOVA for repeated measures, followed by Duncan's multiple-range test. A Students' *t* test was used to detect significant difference between two groups. The statistical analysis was performed by SigmaStat 3.5 software (Systat Software, IL). *P* < 0.05 was considered statistically significant.
